# Crystal structure of 1-bromo-4-methane­sulfonyl-2,3-di­methyl­benzene

**DOI:** 10.1107/S205698901502099X

**Published:** 2015-11-21

**Authors:** Shangwei Dai, Yifeng Wang

**Affiliations:** a Zhejiang Key Laboratory of Green Pesticides and Cleaner Production Technology, Zhejiang University of Technology, Hangzhou 310014, People’s Republic of China

**Keywords:** crystal structure, sulfon­yl, Topramezone, inter­mediate, Br⋯O inter­actions

## Abstract

The title compound, C_9_H_11_BrO_2_S, is an important inter­mediate in the synthesis of the herbicide Topramezone. In the crystal, there are weak inter­molecular Br⋯O inter­actions of 3.286 (4) Å. The dihedral angle between the plane of the benzene ring and that defined by the O—S—O atoms of the methane­sulfonyl group is 49.06 (3)°.

## Related literature   

For general background information, including the synthesis of the title compound, see: Joachim *et al.* (2007 ▸, 2011 ▸).
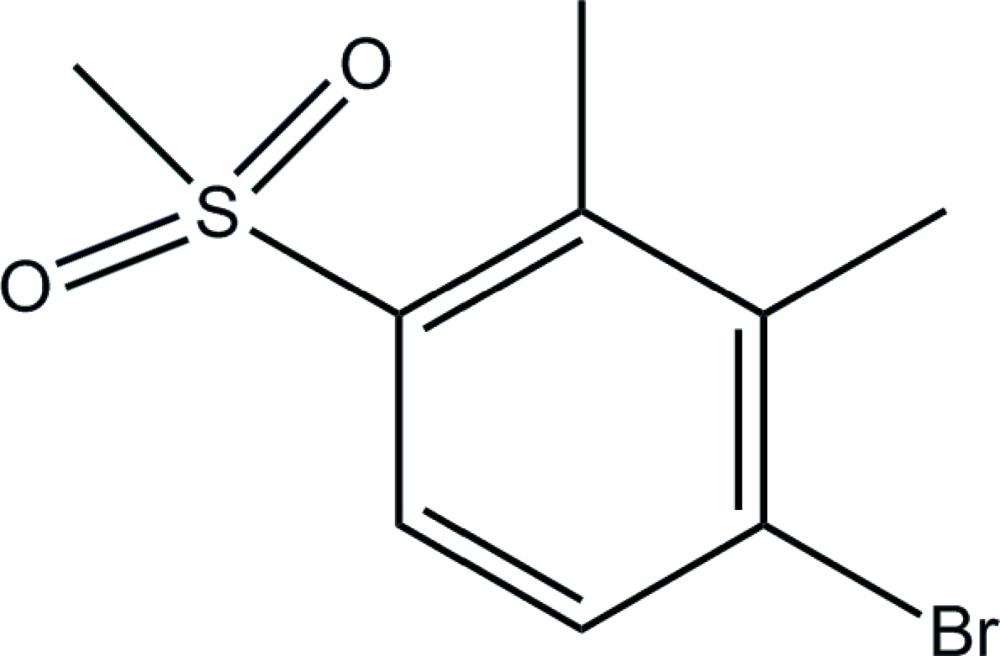


## Experimental   

### Crystal data  

C_9_H_11_BrO_2_S
*M* *_r_* = 263.15
Monoclinic, 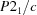

*a* = 8.808 (8) Å
*b* = 5.247 (5) Å
*c* = 22.66 (2) Å
β = 100.956 (15)°
*V* = 1028.0 (16) Å^3^
*Z* = 4
Mo *K*α radiation
μ = 4.17 mm^−1^
*T* = 296 K
0.21 × 0.17 × 0.13 mm

### Data collection  

Bruker APEXII CCD diffractometer
Absorption correction: multi-scan (*SADABS*; Krause *et al.*, 2015 ▸) *T*_min_ = 0.475, *T*_max_ = 0.613
4866 measured reflections
1809 independent reflections
1263 reflections with *I* > 2σ(*I*)
*R*_int_ = 0.030

### Refinement  

*R*[*F* ^2^ > 2σ(*F* ^2^)] = 0.041
*wR*(*F* ^2^) = 0.111
*S* = 1.07
1809 reflections
121 parameters
H-atom parameters constrained
Δρ_max_ = 0.64 e Å^−3^
Δρ_min_ = −0.48 e Å^−3^

### 

Data collection: *APEX2* (Bruker, 2000 ▸); cell refinement: *SAINT* (Bruker, 2000 ▸); data reduction: *SAINT*; program(s) used to solve structure: *SHELXS97* (Sheldrick, 2008 ▸); program(s) used to refine structure: *SHELXL97* (Sheldrick, 2008 ▸); molecular graphics: *XP* in *SHELXTL* (Sheldrick, 2008 ▸); software used to prepare material for publication: *SHELXTL*.

## Supplementary Material

Crystal structure: contains datablock(s) I. DOI: 10.1107/S205698901502099X/pk2567sup1.cif

Structure factors: contains datablock(s) I. DOI: 10.1107/S205698901502099X/pk2567Isup2.hkl

Supporting information file. DOI: 10.1107/S205698901502099X/pk2567Isup3.cml

**Fig. 1.**
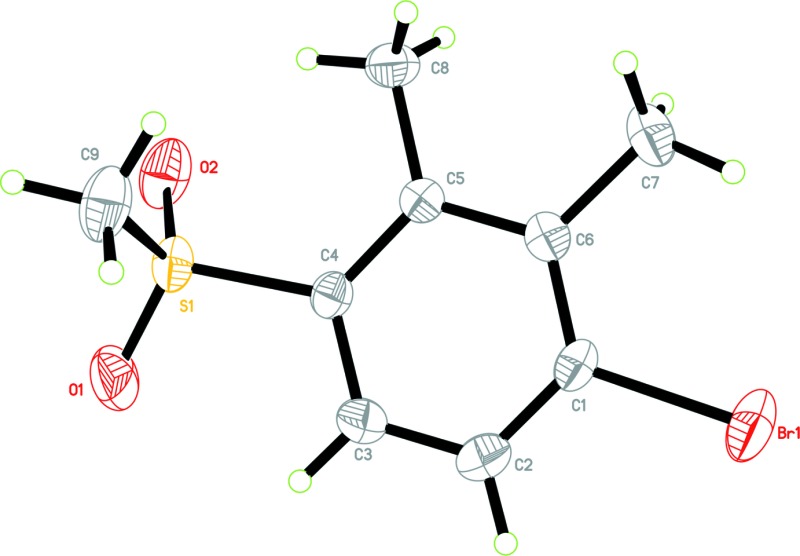
The structure of the title compound. Displacement ellipsoids are drawn at the 50% probability level.

**Fig. 2.**
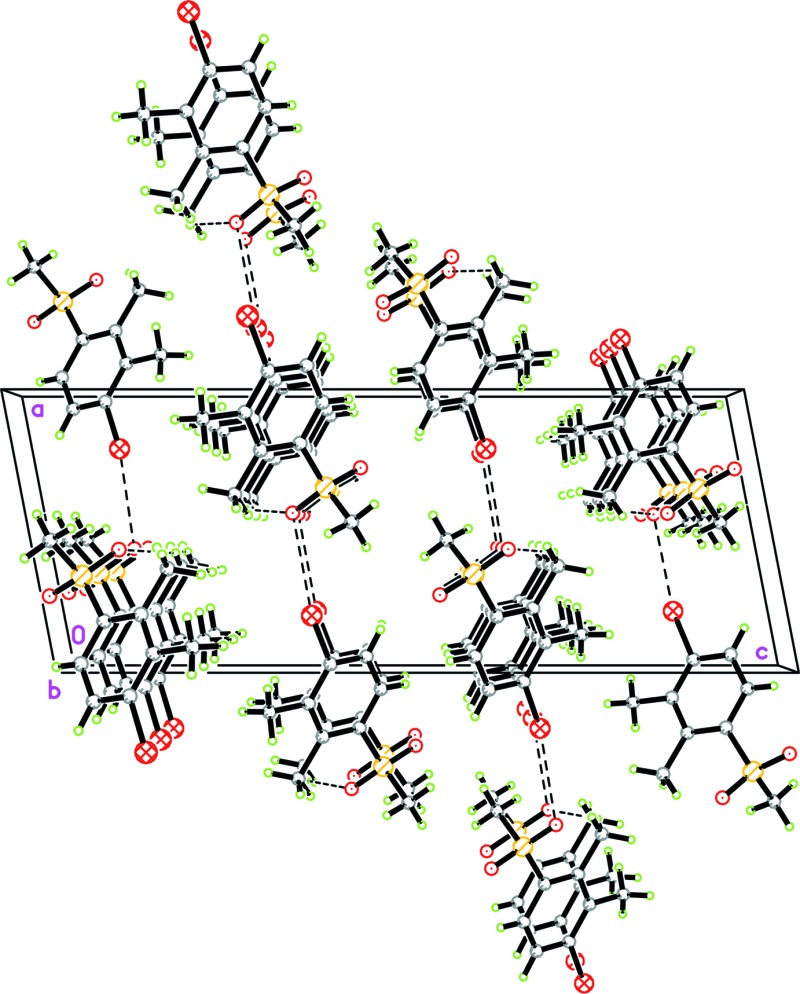
The crystal packing of the title compound viewed down the b axis.

. DOI: 10.1107/S205698901502099X/pk2567fig1.tif
The structure of the title compound. Displacement ellipsoids are drawn at the 50% probability level.

b . DOI: 10.1107/S205698901502099X/pk2567fig2.tif
The crystal packing of the title compound viewed down the *b* axis.

CCDC reference: 1435173

Additional supporting information:  crystallographic information; 3D view; checkCIF report

## supplementary crystallographic information

### S1. Comment

The title compound, C_9_H_11_O_2_SBr, was readily synthesized by the oxidation of 1-bromo-2,3-dimethyl-4-(methylthio)benzene using H_2_O_2_ as the oxidizing agent and Na_2_WO_4_ as catalyst. This compound is an intermediate in the synthesis of Topramezone. In this article, the crystal structure of the title compound is presented (Figs. 1 & 2). In the crystal, there are weak intermolecular Br···O interactions between Br1 and O2 of a symmetry-related [(1 + x, 1 + y, z)] molecule, with a Br···O distance of 3.286 (4) Å. The dihedral angle between the benzene ring and the plane defined by the three atoms (O—S—O) of the methanesulfonyl group is 49.06 (3)°. The bond angle of the O—S—O group is 117.11 (3)°, and the distance between the two oxygen atoms is 2.432 (2) Å.

### S2. Experimental

In a reaction flask, 1-bromo-2,3-dimethyl-4-(methylthio)-benzene (0.03 mol, 4.56 g), and Na_2_WO_4_ (0.68 mmol, 0.20 g) were added in acetic acid (10 ml). The mixture was stirred and heated to 100°C, then H_2_O_2_ (0.09 mol, 10.2 g, 30%) was added dropwise over a period of 1 h. After the reaction was complete (monitored by GC—MS), the mixture was cooled to room temperature and poured into ice water (100 ml) and stirred for 0.5 h, and then filtered. The filtered cake was washed with water (10 ml) and dried to give yellow solid. Single crystals were obtained by slow evaporation of a dichloromethane solution.

### S3. Refinement

All H atoms were placed at calculated positions and allowed to ride on their parent atoms, with C—H = 0.93–0.96 Å and *U*_iso_(H) = 1.2 or 1.5*U*_eq_ (C).

### Crystal data

**Table:**

C_9_H_11_BrO_2_S	*F*(000) = 528
*M**_r_* = 263.15	*D*_x_ = 1.700 Mg m^−^^3^
Monoclinic, *P*2_1_/*c*	Mo *K*α radiation, λ = 0.71073 Å
Hall symbol: -P 2ybc	Cell parameters from 1424 reflections
*a* = 8.808 (8) Å	θ = 2.7–24.0°
*b* = 5.247 (5) Å	µ = 4.17 mm^−^^1^
*c* = 22.66 (2) Å	*T* = 296 K
β = 100.956 (15)°	Block, yellow
*V* = 1028.0 (16) Å^3^	0.21 × 0.17 × 0.13 mm
*Z* = 4	

### Data collection

**Table:**

Bruker APEXII CCD diffractometer	1809 independent reflections
Radiation source: fine-focus sealed tube	1263 reflections with *I* > 2σ(*I*)
Graphite monochromator	*R*_int_ = 0.030
φ and ω scans	θ_max_ = 25.0°, θ_min_ = 1.8°
Absorption correction: multi-scan (*SADABS*; Krause *et al.*, 2015)	*h* = −8→10
*T*_min_ = 0.475, *T*_max_ = 0.613	*k* = −6→6
4866 measured reflections	*l* = −26→26

### Refinement

**Table:**

Refinement on *F*^2^	Primary atom site location: structure-invariant direct methods
Least-squares matrix: full	Secondary atom site location: difference Fourier map
*R*[*F*^2^ > 2σ(*F*^2^)] = 0.041	Hydrogen site location: inferred from neighbouring sites
*wR*(*F*^2^) = 0.111	H-atom parameters constrained
*S* = 1.07	*w* = 1/[σ^2^(*F*_o_^2^) + (0.0594*P*)^2^ + 0.1157*P*] where *P* = (*F*_o_^2^ + 2*F*_c_^2^)/3
1809 reflections	(Δ/σ)_max_ = 0.001
121 parameters	Δρ_max_ = 0.64 e Å^−^^3^
0 restraints	Δρ_min_ = −0.48 e Å^−^^3^

### Special details

**Table:**

Geometry. All e.s.d.'s (except the e.s.d. in the dihedral angle between two l.s. planes) are estimated using the full covariance matrix. The cell e.s.d.'s are taken into account individually in the estimation of e.s.d.'s in distances, angles and torsion angles; correlations between e.s.d.'s in cell parameters are only used when they are defined by crystal symmetry. An approximate (isotropic) treatment of cell e.s.d.'s is used for estimating e.s.d.'s involving l.s. planes.
Refinement. Refinement of *F*^2^ against ALL reflections. The weighted *R*-factor *wR* and goodness of fit *S* are based on *F*^2^, conventional *R*-factors *R* are based on *F*, with *F* set to zero for negative *F*^2^. The threshold expression of *F*^2^ > σ(*F*^2^) is used only for calculating *R*-factors(gt) *etc*. and is not relevant to the choice of reflections for refinement. *R*-factors based on *F*^2^ are statistically about twice as large as those based on *F*, and *R*- factors based on ALL data will be even larger.

### Fractional atomic coordinates and isotropic or equivalent isotropic displacement parameters (Å^2^)

**Table:**

	*x*	*y*	*z*	*U*_iso_*/*U*_eq_	
Br1	1.20567 (5)	1.24921 (9)	0.36104 (2)	0.0774 (3)	
S1	0.64891 (11)	0.56778 (19)	0.41420 (4)	0.0485 (3)	
C4	0.7962 (4)	0.7560 (6)	0.39287 (15)	0.0392 (9)	
C6	0.8938 (4)	1.0673 (7)	0.33261 (14)	0.0391 (8)	
C1	1.0328 (4)	1.0524 (7)	0.37228 (16)	0.0450 (9)	
C5	0.7718 (4)	0.9127 (6)	0.34188 (14)	0.0354 (8)	
O2	0.5621 (3)	0.4403 (6)	0.36349 (13)	0.0748 (9)	
O1	0.7180 (4)	0.4133 (7)	0.46413 (14)	0.0822 (10)	
C9	0.5306 (6)	0.7918 (8)	0.4399 (2)	0.0679 (13)	
H9A	0.4920	0.9096	0.4082	0.102*	
H9B	0.5893	0.8827	0.4734	0.102*	
H9C	0.4455	0.7065	0.4523	0.102*	
C8	0.6225 (4)	0.9180 (8)	0.29757 (16)	0.0528 (10)	
H8A	0.5540	0.7908	0.3081	0.079*	
H8B	0.6418	0.8837	0.2580	0.079*	
H8C	0.5757	1.0831	0.2981	0.079*	
C3	0.9363 (5)	0.7514 (7)	0.43240 (18)	0.0551 (11)	
H3	0.9490	0.6483	0.4664	0.066*	
C2	1.0550 (4)	0.8967 (9)	0.42176 (19)	0.0601 (11)	
H2	1.1505	0.8910	0.4477	0.072*	
C7	0.8704 (6)	1.2484 (7)	0.28001 (19)	0.0602 (12)	
H7A	0.9538	1.3692	0.2852	0.090*	
H7B	0.7742	1.3373	0.2778	0.090*	
H7C	0.8683	1.1543	0.2435	0.090*	

### Atomic displacement parameters (Å^2^)

**Table:**

	*U*^11^	*U*^22^	*U*^33^	*U*^12^	*U*^13^	*U*^23^
Br1	0.0506 (3)	0.0762 (4)	0.1110 (5)	−0.0200 (2)	0.0293 (3)	0.0004 (3)
S1	0.0482 (5)	0.0414 (6)	0.0613 (6)	−0.0039 (4)	0.0240 (5)	0.0013 (5)
C4	0.0371 (19)	0.041 (2)	0.042 (2)	−0.0012 (16)	0.0123 (16)	−0.0033 (17)
C6	0.045 (2)	0.035 (2)	0.0399 (19)	0.0051 (17)	0.0152 (16)	−0.0030 (17)
C1	0.0328 (19)	0.047 (2)	0.057 (2)	−0.0052 (16)	0.0142 (17)	−0.002 (2)
C5	0.0373 (18)	0.0315 (19)	0.0385 (19)	0.0038 (16)	0.0096 (15)	−0.0045 (16)
O2	0.0742 (19)	0.065 (2)	0.090 (2)	−0.0315 (16)	0.0290 (17)	−0.0260 (18)
O1	0.082 (2)	0.077 (2)	0.093 (2)	0.0011 (18)	0.0300 (18)	0.0428 (19)
C9	0.064 (3)	0.061 (3)	0.090 (3)	0.000 (2)	0.044 (3)	−0.011 (2)
C8	0.047 (2)	0.053 (3)	0.053 (2)	0.0019 (19)	−0.0024 (18)	0.002 (2)
C3	0.047 (2)	0.064 (3)	0.052 (2)	0.005 (2)	0.0045 (19)	0.017 (2)
C2	0.037 (2)	0.073 (3)	0.066 (3)	0.001 (2)	0.0010 (19)	0.009 (2)
C7	0.076 (3)	0.047 (3)	0.063 (3)	0.001 (2)	0.026 (2)	0.013 (2)

### Geometric parameters (Å, º)

**Table:**

Br1—C1	1.896 (4)	C9—H9A	0.9600
S1—O2	1.421 (3)	C9—H9B	0.9600
S1—O1	1.430 (3)	C9—H9C	0.9600
S1—C9	1.743 (4)	C8—H8A	0.9600
S1—C4	1.770 (3)	C8—H8B	0.9600
C4—C5	1.401 (5)	C8—H8C	0.9600
C4—C3	1.380 (5)	C3—C2	1.352 (5)
C6—C1	1.377 (5)	C3—H3	0.9300
C6—C5	1.393 (5)	C2—H2	0.9300
C6—C7	1.508 (5)	C7—H7A	0.9600
C1—C2	1.371 (5)	C7—H7B	0.9600
C5—C8	1.496 (5)	C7—H7C	0.9600
			
O2—S1—O1	117.1 (2)	S1—C9—H9C	109.5
O2—S1—C9	108.9 (2)	H9A—C9—H9C	109.5
O1—S1—C9	108.0 (2)	H9B—C9—H9C	109.5
O2—S1—C4	110.59 (17)	C5—C8—H8A	109.5
O1—S1—C4	107.95 (19)	C5—C8—H8B	109.5
C9—S1—C4	103.3 (2)	H8A—C8—H8B	109.5
C5—C4—C3	121.6 (3)	C5—C8—H8C	109.5
C5—C4—S1	123.2 (3)	H8A—C8—H8C	109.5
C3—C4—S1	115.1 (3)	H8B—C8—H8C	109.5
C1—C6—C5	119.0 (3)	C2—C3—C4	120.0 (4)
C1—C6—C7	121.5 (3)	C2—C3—H3	120.0
C5—C6—C7	119.5 (3)	C4—C3—H3	120.0
C6—C1—C2	122.6 (3)	C3—C2—C1	119.1 (4)
C6—C1—Br1	121.2 (3)	C3—C2—H2	120.4
C2—C1—Br1	116.2 (3)	C1—C2—H2	120.4
C4—C5—C6	117.6 (3)	C6—C7—H7A	109.5
C4—C5—C8	122.8 (3)	C6—C7—H7B	109.5
C6—C5—C8	119.5 (3)	H7A—C7—H7B	109.5
S1—C9—H9A	109.5	C6—C7—H7C	109.5
S1—C9—H9B	109.5	H7A—C7—H7C	109.5
H9A—C9—H9B	109.5	H7B—C7—H7C	109.5
			
O2—S1—C4—C5	44.6 (3)	C3—C4—C5—C8	178.7 (4)
O1—S1—C4—C5	173.9 (3)	S1—C4—C5—C8	−5.7 (4)
C9—S1—C4—C5	−71.8 (3)	C1—C6—C5—C4	2.3 (5)
O2—S1—C4—C3	−139.5 (3)	C7—C6—C5—C4	−177.0 (3)
O1—S1—C4—C3	−10.2 (3)	C1—C6—C5—C8	−177.2 (3)
C9—S1—C4—C3	104.1 (3)	C7—C6—C5—C8	3.5 (5)
C5—C6—C1—C2	−2.0 (5)	C5—C4—C3—C2	−1.3 (6)
C7—C6—C1—C2	177.3 (4)	S1—C4—C3—C2	−177.3 (3)
C5—C6—C1—Br1	178.0 (2)	C4—C3—C2—C1	1.7 (6)
C7—C6—C1—Br1	−2.7 (5)	C6—C1—C2—C3	−0.1 (6)
C3—C4—C5—C6	−0.7 (5)	Br1—C1—C2—C3	180.0 (3)
S1—C4—C5—C6	174.9 (2)		
