# Supplementary figures and images for: Crystal structure of 1-bromo-4-methane­sulfonyl-2,3-di­methyl­benzene
Source: Acta Crystallogr E Crystallogr Commun. 2015 Nov 21;71(Pt 12):o973. doi: 10.1107/S205698901502099X (PMC4719929; doi:10.1107/S205698901502099X)

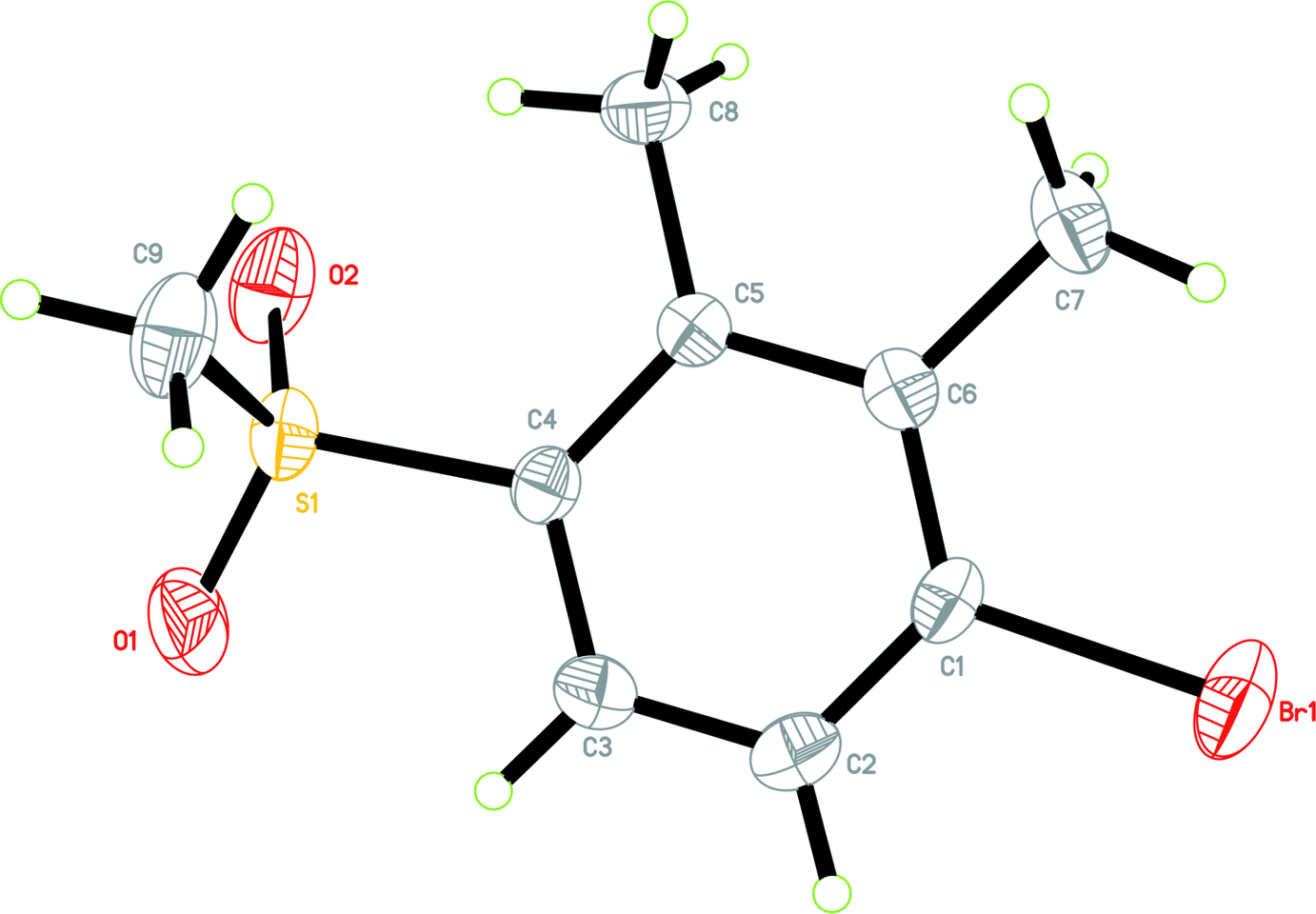

Supplement: Supplementary file 4 [file e-71-0o973-fig1.tif]

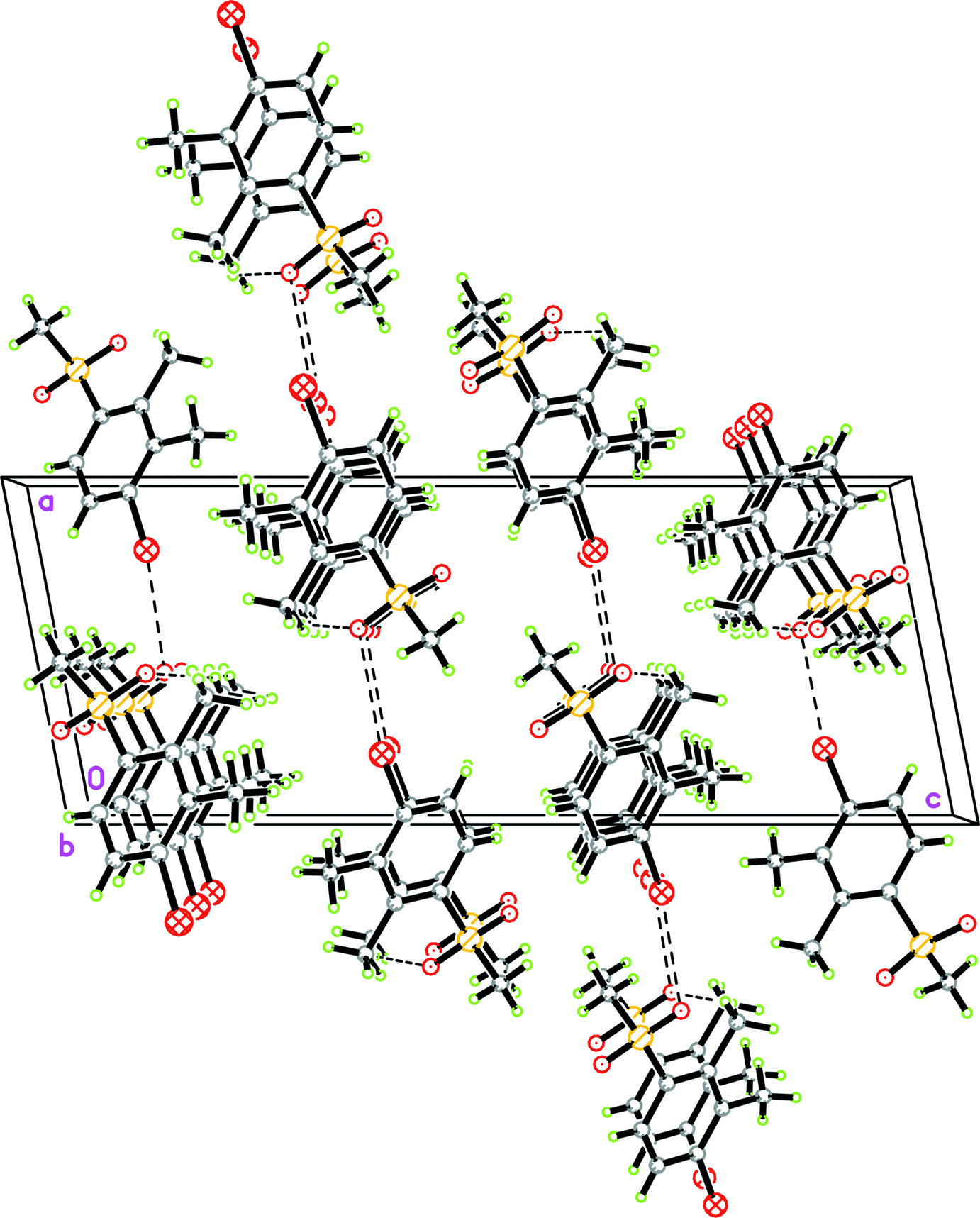

Supplement: Supplementary file 5 [file e-71-0o973-fig2.tif]
